# 1-[Bis(4-fluoro­phen­yl)meth­yl]-4-[(2*Z*)-3-phenyl­prop-2-en-1-yl]piperazine-1,4-diium dichloride hemihydrate

**DOI:** 10.1107/S1600536814011064

**Published:** 2014-05-21

**Authors:** S. Shivaprakash, G. Chandrasekara Reddy, Jerry P. Jasinski

**Affiliations:** a Vittal Mallya Scientific Research Foundation, #94/3, 23rd Cross; 29th Main; BTM II Stage, Bangalore, 560-076, India; b Department of Chemistry, Keene State College, 229 Main Street, Keene, NH 03435-2001, USA

## Abstract

The asymmetric unit of the title monohydrated salt, 2C_26_H_28_F_2_N_2_^2+^·4Cl^−.^H_2_O, consists of a 1-[bis­(4-fluoro­phen­yl)meth­yl]-4-[(2*Z*)-3-phenyl­prop-2-en-1-yl]piperazine-1,4-diium cation with a diprotonated piperizine ring in close proximity to two chloride anions and a single water mol­ecule that lies on a twofold rotation axis. In the cation, the piperazine ring adopts a slightly distorted chair conformation. The dihedral angles between the phenyl ring and the 4-fluoro­phenyl rings are 89.3 (9) and 35.0 (5)°. The two fluoro­phenyl rings are inclined at 65.0 (5)° to one another. In the crystal, N—H⋯Cl hydrogen bonds and weak C—H⋯Cl inter­molecular inter­actions link the mol­ecules into chains along [010]. In addition, weak C—H⋯O inter­actions between the piperizine and prop-2-en-1-yl groups with the water mol­ecule, along with weak C—H⋯Cl inter­actions between the prop-2en-1-yl and methyl groups with the chloride ions, weak C—H⋯F inter­actions between the two fluoro­phenyl groups and weak O—H⋯Cl inter­actions between the water mol­ecule and chloride ions form a three-dimensional supra­molecular network.

## Related literature   

For the use of flunarizine {systematic name: (*E*)-1-[bis­(4-fluoro­phen­yl)meth­yl]-4-(3-phenyl-2-propen­yl)piperazine} as an anti­histamine and vasodilator, see: Agnoli *et al.* (1988 ▶); Prasanna & Row (2001 ▶). For the synthesis of (*E*)-isomers of 1-benzhydryl-4-cinnamyl piperazines, see: Cignarella & Testa (1968 ▶) and that of the Z-isomer of cinnerizine, [systematic name: (*E*)-1-(di­phenyl­meth­yl)-4-(3-phenyl­prop-2-en­yl)piper­azine], see; Shivaprakash & Chandrasekara Reddy (2014 ▶). For puckering parameters, see Cremer & Pople (1975 ▶). For standard bond lengths, see: Allen *et al.* (1987 ▶).
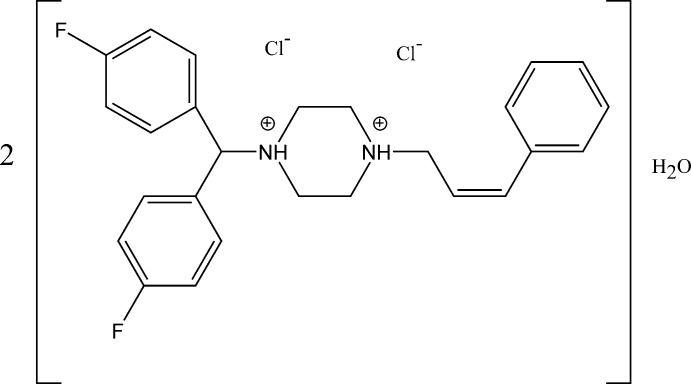


## Experimental   

### 

#### Crystal data  

2C_26_H_28_F_2_N_2_^2+^·4Cl^−^·H_2_O
*M* *_r_* = 972.82
Monoclinic, 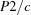

*a* = 18.2973 (6) Å
*b* = 7.02041 (14) Å
*c* = 20.1554 (6) Å
β = 104.601 (3)°
*V* = 2505.44 (13) Å^3^
*Z* = 2
Mo *K*α radiation
μ = 0.29 mm^−1^
*T* = 173 K
0.44 × 0.38 × 0.16 mm

#### Data collection  

Agilent Eos Gemini diffractometer
Absorption correction: multi-scan (*CrysAlis PRO* and *CrysAlis RED*; Agilent, 2012 ▶) *T*_min_ = 0.914, *T*_max_ = 1.000
32975 measured reflections
8607 independent reflections
6582 reflections with *I* > 2σ(*I*)
*R*_int_ = 0.034

#### Refinement  

*R*[*F* ^2^ > 2σ(*F* ^2^)] = 0.057
*wR*(*F* ^2^) = 0.153
*S* = 1.09
8607 reflections
306 parameters
H atoms treated by a mixture of independent and constrained refinement
Δρ_max_ = 0.65 e Å^−3^
Δρ_min_ = −0.41 e Å^−3^

### 

Data collection: *CrysAlis PRO* (Agilent, 2012 ▶); cell refinement: *CrysAlis PRO*; data reduction: *CrysAlis RED* (Agilent, 2012 ▶); program(s) used to solve structure: *SUPERFLIP* (Palatinus & Chapuis, 2007 ▶; Palatinus & van der Lee, 2008 ▶; Palatinus *et al.*, 2012 ▶); program(s) used to refine structure: *SHELXL2012* (Sheldrick, 2008 ▶); molecular graphics: *OLEX2* (Dolomanov *et al.*, 2009 ▶); software used to prepare material for publication: *OLEX2*.

## Supplementary Material

Crystal structure: contains datablock(s) I. DOI: 10.1107/S1600536814011064/sj5400sup1.cif

Structure factors: contains datablock(s) I. DOI: 10.1107/S1600536814011064/sj5400Isup2.hkl

Supporting information file. DOI: 10.1107/S1600536814011064/sj5400Isup3.cml

CCDC reference: 1002927

Additional supporting information:  crystallographic information; 3D view; checkCIF report

## Figures and Tables

**Table 1:** Hydrogen-bond geometry (Å, °)

*D*—H⋯*A*	*D*—H	H⋯*A*	*D*⋯*A*	*D*—H⋯*A*
N1—H1⋯Cl1^i^	0.84 (2)	2.14 (2)	2.9782 (13)	176 (2)
N2—H2⋯Cl2^ii^	0.86 (3)	2.17 (3)	3.0248 (13)	174 (2)
C1—H1*A*⋯O1*W* ^i^	0.99	2.68	3.512 (2)	142
C5—H5*A*⋯O1*W* ^i^	0.99	2.67	3.470 (2)	138
C5—H5*B*⋯Cl1^ii^	0.99	2.51	3.4934 (16)	176
C14—H14⋯Cl2^i^	1.00	2.49	3.4735 (15)	168
C19—H19⋯F2^iii^	0.95	2.46	3.244 (2)	140
O1*W*—H1*W*⋯Cl1	0.80 (3)	2.48 (3)	3.2603 (12)	163 (3)

Symmetry codes: (i) 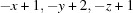; (ii) 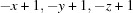; (iii) 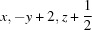.

**Fig. 1.**
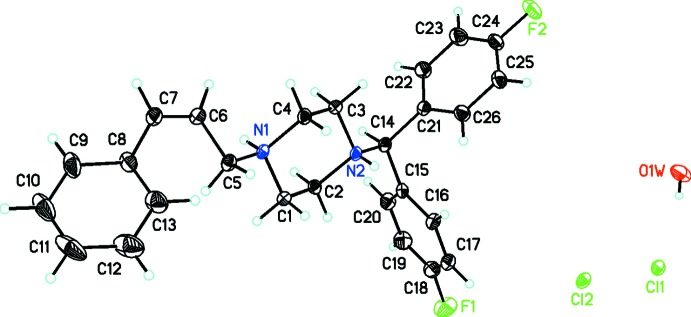
ORTEP drawing of the asymmetric unit of (I), showing the labeling scheme and with 30% probability displacement ellipsoids.

**Fig. 2.**
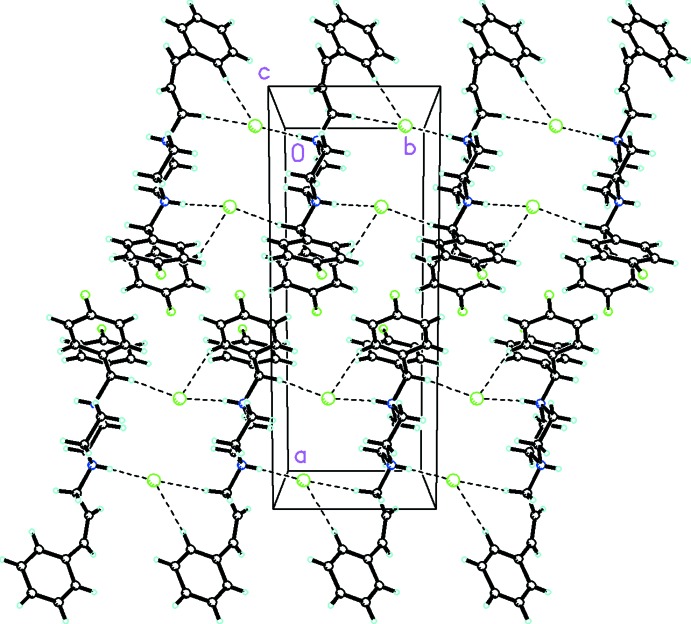
Crystal packing of (I) viewed along the c axis. Dashed lines indicate N—H···Cl hydrogen bonds and weak C—H···Cl intermolecular interactions which link the molecules into chains along [010].

## supplementary crystallographic information

### 1. Comment

Flunarizine, (E)-1-[bis(4-fluorophenyl)methyl]-4-(3-phenyl-2- propenyl)piperazine, is a clinically useful drug used as an antihistamine and vasodilator (Agnoli *et al.* (1988), Prasanna & Row (2001). Because the greater biological importance of (E)-isomers of 1-benzhydryl-4-cinnamyl piperazines has been recognised, several synthetic methods for these isomers have been described (Cignarella & Testa, 1968). However, the synthesis of (Z)-1-[bis(4-fluorophenyl)methyl]-4-(3-phenyl-2-propenyl)piperazine has only recently been reported (Shivaprakash & Chandrasekara Reddy, 2014).

The title compound, 2(C_26_H_28_F_2_N_2_), 4(Cl), H_2_O, (I), is a close analogue of the existing drug, flunarizine, which has an (E) configuration. The crystal structure of flunarizine has been reported (Prasanna & Row, 2001). We have prepared the (Z) isomer (I) for the first time as the crystalline hydrochloride salt to study structure activity relationships and we report its structure here.

The asymmetric unit of the title monohydrated salt, 2(C_26_H_28_F_2_N_2_^2+^), 4(Cl^-^), H_2_O, (I), consists of a [(Z)-1-[bis(4-fluorophenyl)methyl]-4-(3-phenyl-2-propenyl)piperazine] cation with a diprotonated piperizine ring in close proximity to two chloride anions and a single water molecule that lies on a two-fold rotation axis (Fig. 1). The piperazine group adopts a slightly distorted chair conformation (puckering parameters Q, θ, and φ = 0.5911 (15)Å, 0.95 (15)° and 154 (8)°, respectively (Cremer & Pople, 1975). Bond lengths are within normal ranges (Allen *et al.*, 1987). In the cation, the dihedral angles between the mean planes of the two 4-fluorophenyl rings with that of the phenyl ring are 89.3 (9)° and 35.0 (5)°, respectively. The two fluorophenyl groups are inclined at 65.0 (5)° to one another. In the crystal, N—H···Cl hydrogen bonds and weak C—H···Cl intermolecular interactions link the molecules into chains along [010] (Fig. 2). In addition, weak C—H···O interactions between the piperizine and prop-2-en-1-yl groups with the water molecule along with weak C—H···Cl interactions between the prop-2en-1-yl and methyl groups with the chloride ions and weak C—H···F interactions between the two fluorophenyl groups and weak O—H···Cl interactions between the water molecule and chloride ions (Table 1) form a 3-D supramolecular network.

### 2. Experimental

To a solution of 1-(4, 4'-difluoro phenylmethyl)-4-(2-acetaldehyde) piperazine (5.6 g, 17.0 mmol) in dichloromethane (50 ml) under a nitrogen atmosphere was added benzyltriphenyl phosphonium chloride, (6.9 g, 17.9 mmol). The mixture was cooled to 278 °K and t-BuOK (4.6 g, 41.3 mmol) was added with stirring. After completion, the reaction mass was quenched into water (100 ml). The organic layer was separated, dried over anhydrous sodium sulfate and concentrated under vacuum. The solution was then subjected to column chromatography over silica gel with an EtOAc/Hexane (1:9) elutant mixture to afford (Z)-1-[bis-(4-fluorophenyl)-methyl]-4-(cinnamyl) piperazine as a viscous liquid. This was then converted into the hydrochloride salt using ethanolic HCl and crystallized from acetone/ethanol (2:8) mp 473-475°K.

### 3. Refinement

The H1, H2 and H1W atoms were located from a difference map and refined isotropically. All of the remaining H atoms were placed in their calculated positions and then refined using a riding model with Atom—H lengths of 0.95Å (CH) or 0.99Å (CH_2_). Isotropic displacement parameters for these atoms were set to 1.2 (CH, CH_2_) times *U*_eq_ of the parent atom.

### Crystal data

**Table:**

2C_26_H_28_F_2_N_2_^2+^·4Cl^−^·H_2_O	*F*(000) = 1020
*M**_r_* = 972.82	*D*_x_ = 1.290 Mg m^−^^3^
Monoclinic, *P*2/*c*	Mo *K*α radiation, λ = 0.71073 Å
*a* = 18.2973 (6) Å	Cell parameters from 7981 reflections
*b* = 7.02041 (14) Å	θ = 3.5–32.4°
*c* = 20.1554 (6) Å	µ = 0.29 mm^−^^1^
β = 104.601 (3)°	*T* = 173 K
*V* = 2505.44 (13) Å^3^	Irregular, colourless
*Z* = 2	0.44 × 0.38 × 0.16 mm

### Data collection

**Table:**

Agilent Eos Gemini diffractometer	8607 independent reflections
Radiation source: Enhance (Mo) X-ray Source	6582 reflections with *I* > 2σ(*I*)
Detector resolution: 16.0416 pixels mm^-1^	*R*_int_ = 0.034
ω scans	θ_max_ = 32.8°, θ_min_ = 3.1°
Absorption correction: multi-scan (*CrysAlis PRO* and *CrysAlis RED*; Agilent, 2012)	*h* = −27→26
*T*_min_ = 0.914, *T*_max_ = 1.000	*k* = −10→10
32975 measured reflections	*l* = −29→29

### Refinement

**Table:**

Refinement on *F*^2^	Primary atom site location: structure-invariant direct methods
Least-squares matrix: full	Hydrogen site location: mixed
*R*[*F*^2^ > 2σ(*F*^2^)] = 0.057	H atoms treated by a mixture of independent and constrained refinement
*wR*(*F*^2^) = 0.153	*w* = 1/[σ^2^(*F*_o_^2^) + (0.0652*P*)^2^ + 1.0043*P*] where *P* = (*F*_o_^2^ + 2*F*_c_^2^)/3
*S* = 1.09	(Δ/σ)_max_ = 0.001
8607 reflections	Δρ_max_ = 0.65 e Å^−^^3^
306 parameters	Δρ_min_ = −0.41 e Å^−^^3^
0 restraints	

### Special details

**Table:**

Experimental. ^1^H NMR: δ 7.20 - 7.35(m, 9 H, Ar-H), 6.87-6.98 (m, 4 H, Ar-H), 6.50 (d, J = 12 Hz, 1 H), 5.76 (ddd, J = 12.0, 6.6 Hz, 1 H), 4.20(s, 1 H), 3.27 (dd, J = 6.6, 1.8 Hz, 2 H), 2.40 (bd, 8 H). ^13^C NMR: δ 163.1, 160.6, 138.3, 137.1, 132.6, 132.5, 131.5, 129.3, 129.2, 128.9, 128.2, 126.9, 115.5, 115.3, 74.5, 56.1, 53.4, 51.7. HRMS calculated for C_26_H_26_F_2_N_2_ [M+H] + 405.2142; found 405.2145.
Geometry. All esds (except the esd in the dihedral angle between two l.s. planes) are estimated using the full covariance matrix. The cell esds are taken into account individually in the estimation of esds in distances, angles and torsion angles; correlations between esds in cell parameters are only used when they are defined by crystal symmetry. An approximate (isotropic) treatment of cell esds is used for estimating esds involving l.s. planes.

### Fractional atomic coordinates and isotropic or equivalent isotropic displacement parameters (Å^2^)

**Table:**

	*x*	*y*	*z*	*U*_iso_*/*U*_eq_	
F1	0.47811 (8)	0.7591 (2)	0.63602 (8)	0.0647 (4)	
F2	0.57160 (8)	0.7045 (2)	0.20291 (6)	0.0572 (3)	
N1	0.92882 (7)	0.74830 (17)	0.58339 (6)	0.0225 (2)	
H1	0.9389 (11)	0.865 (3)	0.5856 (10)	0.032 (5)*	
N2	0.76733 (7)	0.75240 (17)	0.51554 (6)	0.0219 (2)	
H2	0.7600 (14)	0.632 (4)	0.5145 (13)	0.054 (7)*	
C1	0.87041 (8)	0.7138 (2)	0.62188 (8)	0.0263 (3)	
H1A	0.8893	0.7589	0.6697	0.032*	
H1B	0.8603	0.5754	0.6231	0.032*	
C2	0.79796 (8)	0.8174 (2)	0.58809 (7)	0.0253 (3)	
H2A	0.7599	0.7938	0.6144	0.030*	
H2B	0.8078	0.9562	0.5886	0.030*	
C3	0.82618 (8)	0.7885 (2)	0.47723 (8)	0.0274 (3)	
H3A	0.8359	0.9271	0.4762	0.033*	
H3B	0.8075	0.7440	0.4293	0.033*	
C4	0.89918 (9)	0.6860 (2)	0.51080 (8)	0.0279 (3)	
H4A	0.8901	0.5468	0.5095	0.033*	
H4B	0.9373	0.7127	0.4848	0.033*	
C5	1.00129 (8)	0.6496 (2)	0.61791 (8)	0.0280 (3)	
H5A	1.0139	0.6773	0.6677	0.034*	
H5B	0.9946	0.5102	0.6118	0.034*	
C6	1.06496 (9)	0.7136 (2)	0.58886 (9)	0.0308 (3)	
H6	1.0524	0.7780	0.5460	0.037*	
C7	1.13753 (10)	0.6868 (3)	0.61864 (9)	0.0366 (4)	
H7	1.1721	0.7381	0.5952	0.044*	
C8	1.17121 (9)	0.5878 (3)	0.68320 (9)	0.0388 (4)	
C9	1.23672 (11)	0.6637 (5)	0.72635 (12)	0.0609 (7)	
H9	1.2568	0.7796	0.7142	0.073*	
C10	1.27239 (13)	0.5731 (6)	0.78600 (13)	0.0792 (11)	
H10	1.3171	0.6264	0.8145	0.095*	
C11	1.24474 (16)	0.4094 (6)	0.80465 (12)	0.0829 (12)	
H11	1.2700	0.3480	0.8461	0.100*	
C12	1.17920 (16)	0.3301 (5)	0.76321 (14)	0.0731 (8)	
H12	1.1592	0.2155	0.7765	0.088*	
C13	1.14318 (12)	0.4205 (4)	0.70195 (11)	0.0517 (5)	
H13	1.0990	0.3659	0.6731	0.062*	
C14	0.69337 (8)	0.8548 (2)	0.48296 (8)	0.0251 (3)	
H14	0.7049	0.9942	0.4854	0.030*	
C15	0.63586 (8)	0.8223 (2)	0.52455 (8)	0.0257 (3)	
C16	0.60876 (9)	0.6424 (2)	0.53524 (9)	0.0308 (3)	
H16	0.6271	0.5335	0.5165	0.037*	
C17	0.55524 (9)	0.6199 (3)	0.57288 (9)	0.0357 (4)	
H17	0.5370	0.4972	0.5805	0.043*	
C18	0.52962 (10)	0.7797 (3)	0.59865 (10)	0.0396 (4)	
C19	0.55408 (11)	0.9604 (3)	0.58915 (11)	0.0439 (4)	
H19	0.5348	1.0684	0.6075	0.053*	
C20	0.60802 (10)	0.9802 (3)	0.55179 (9)	0.0355 (4)	
H20	0.6261	1.1035	0.5448	0.043*	
C21	0.66394 (8)	0.8064 (2)	0.40760 (8)	0.0263 (3)	
C22	0.65150 (11)	0.9551 (3)	0.36112 (9)	0.0367 (4)	
H22	0.6645	1.0812	0.3768	0.044*	
C23	0.62021 (12)	0.9224 (3)	0.29177 (10)	0.0463 (5)	
H23	0.6114	1.0246	0.2599	0.056*	
C24	0.60250 (10)	0.7394 (3)	0.27056 (9)	0.0378 (4)	
C25	0.61411 (11)	0.5880 (3)	0.31451 (9)	0.0401 (4)	
H25	0.6013	0.4624	0.2982	0.048*	
C26	0.64509 (11)	0.6225 (3)	0.38353 (9)	0.0374 (4)	
H26	0.6536	0.5191	0.4149	0.045*	
Cl2	0.24638 (3)	0.67703 (5)	0.48578 (2)	0.03563 (11)	
Cl1	0.03137 (3)	0.83990 (6)	0.40147 (2)	0.04480 (13)	
O1W	0.0000	1.0343 (4)	0.2500	0.0721 (9)	
H1W	0.009 (2)	0.965 (5)	0.2828 (16)	0.095 (12)*	

### Atomic displacement parameters (Å^2^)

**Table:**

	*U*^11^	*U*^22^	*U*^33^	*U*^12^	*U*^13^	*U*^23^
F1	0.0528 (8)	0.0800 (10)	0.0764 (10)	0.0008 (7)	0.0443 (7)	−0.0034 (8)
F2	0.0648 (8)	0.0718 (9)	0.0270 (5)	−0.0031 (7)	−0.0032 (5)	−0.0023 (6)
N1	0.0233 (6)	0.0167 (5)	0.0277 (6)	−0.0048 (4)	0.0065 (4)	−0.0019 (4)
N2	0.0232 (6)	0.0172 (5)	0.0252 (6)	−0.0048 (4)	0.0057 (4)	−0.0032 (4)
C1	0.0257 (7)	0.0268 (7)	0.0268 (7)	−0.0042 (5)	0.0074 (5)	0.0011 (5)
C2	0.0268 (7)	0.0246 (7)	0.0242 (7)	−0.0031 (5)	0.0059 (5)	−0.0049 (5)
C3	0.0259 (7)	0.0326 (7)	0.0246 (7)	−0.0066 (6)	0.0079 (5)	−0.0016 (6)
C4	0.0269 (7)	0.0286 (7)	0.0291 (7)	−0.0058 (6)	0.0087 (5)	−0.0083 (6)
C5	0.0255 (7)	0.0218 (6)	0.0354 (8)	−0.0002 (5)	0.0055 (6)	0.0035 (6)
C6	0.0285 (7)	0.0307 (7)	0.0337 (8)	−0.0028 (6)	0.0084 (6)	0.0004 (6)
C7	0.0276 (8)	0.0473 (10)	0.0362 (9)	−0.0054 (7)	0.0105 (6)	−0.0044 (7)
C8	0.0229 (7)	0.0622 (12)	0.0310 (8)	0.0065 (7)	0.0065 (6)	−0.0085 (8)
C9	0.0273 (9)	0.109 (2)	0.0442 (11)	−0.0009 (11)	0.0045 (8)	−0.0229 (12)
C10	0.0341 (11)	0.163 (3)	0.0369 (12)	0.0208 (16)	0.0014 (9)	−0.0149 (16)
C11	0.0559 (15)	0.161 (3)	0.0311 (11)	0.0581 (19)	0.0092 (10)	0.0130 (15)
C12	0.0650 (16)	0.096 (2)	0.0596 (15)	0.0335 (15)	0.0176 (12)	0.0297 (15)
C13	0.0427 (11)	0.0628 (14)	0.0455 (11)	0.0125 (10)	0.0034 (8)	0.0102 (10)
C14	0.0268 (7)	0.0187 (6)	0.0291 (7)	−0.0030 (5)	0.0058 (5)	−0.0020 (5)
C15	0.0232 (6)	0.0260 (7)	0.0261 (7)	−0.0029 (5)	0.0027 (5)	−0.0045 (5)
C16	0.0276 (7)	0.0285 (7)	0.0372 (8)	−0.0037 (6)	0.0098 (6)	−0.0039 (6)
C17	0.0278 (8)	0.0412 (9)	0.0381 (9)	−0.0067 (7)	0.0085 (6)	−0.0017 (7)
C18	0.0268 (8)	0.0566 (11)	0.0378 (9)	−0.0006 (8)	0.0126 (7)	−0.0026 (8)
C19	0.0438 (10)	0.0455 (11)	0.0462 (11)	0.0061 (8)	0.0185 (8)	−0.0103 (8)
C20	0.0388 (9)	0.0285 (8)	0.0397 (9)	−0.0006 (7)	0.0110 (7)	−0.0071 (7)
C21	0.0253 (7)	0.0257 (7)	0.0277 (7)	−0.0024 (5)	0.0060 (5)	−0.0008 (5)
C22	0.0427 (9)	0.0303 (8)	0.0351 (9)	−0.0008 (7)	0.0059 (7)	0.0031 (7)
C23	0.0562 (12)	0.0420 (10)	0.0351 (9)	0.0020 (9)	0.0011 (8)	0.0093 (8)
C24	0.0342 (8)	0.0521 (11)	0.0245 (7)	−0.0011 (8)	0.0023 (6)	−0.0019 (7)
C25	0.0479 (10)	0.0377 (9)	0.0326 (9)	−0.0091 (8)	0.0064 (7)	−0.0079 (7)
C26	0.0532 (11)	0.0284 (8)	0.0285 (8)	−0.0073 (7)	0.0063 (7)	−0.0013 (6)
Cl2	0.0432 (2)	0.01969 (17)	0.0464 (2)	−0.00479 (15)	0.01574 (17)	−0.00344 (15)
Cl1	0.0689 (3)	0.02108 (18)	0.0417 (2)	−0.01358 (19)	0.0089 (2)	−0.00149 (15)
O1W	0.137 (3)	0.0383 (12)	0.0386 (13)	0.000	0.0176 (15)	0.000

### Geometric parameters (Å, º)

**Table:**

F1—C18	1.354 (2)	C10—H10	0.9500
F2—C24	1.360 (2)	C10—C11	1.346 (5)
N1—H1	0.84 (2)	C11—H11	0.9500
N1—C1	1.4903 (19)	C11—C12	1.393 (5)
N1—C4	1.4920 (19)	C12—H12	0.9500
N1—C5	1.5023 (19)	C12—C13	1.397 (3)
N2—H2	0.86 (3)	C13—H13	0.9500
N2—C2	1.4992 (18)	C14—H14	1.0000
N2—C3	1.4962 (19)	C14—C15	1.519 (2)
N2—C14	1.5268 (19)	C14—C21	1.517 (2)
C1—H1A	0.9900	C15—C16	1.394 (2)
C1—H1B	0.9900	C15—C20	1.389 (2)
C1—C2	1.515 (2)	C16—H16	0.9500
C2—H2A	0.9900	C16—C17	1.391 (2)
C2—H2B	0.9900	C17—H17	0.9500
C3—H3A	0.9900	C17—C18	1.368 (3)
C3—H3B	0.9900	C18—C19	1.374 (3)
C3—C4	1.518 (2)	C19—H19	0.9500
C4—H4A	0.9900	C19—C20	1.391 (3)
C4—H4B	0.9900	C20—H20	0.9500
C5—H5A	0.9900	C21—C22	1.383 (2)
C5—H5B	0.9900	C21—C26	1.391 (2)
C5—C6	1.499 (2)	C22—H22	0.9500
C6—H6	0.9500	C22—C23	1.390 (3)
C6—C7	1.326 (2)	C23—H23	0.9500
C7—H7	0.9500	C23—C24	1.366 (3)
C7—C8	1.467 (3)	C24—C25	1.366 (3)
C8—C9	1.397 (3)	C25—H25	0.9500
C8—C13	1.372 (3)	C25—C26	1.385 (2)
C9—H9	0.9500	C26—H26	0.9500
C9—C10	1.371 (4)	O1W—H1W	0.80 (3)
			
C1—N1—H1	108.0 (14)	C9—C10—H10	119.6
C1—N1—C4	109.37 (11)	C11—C10—C9	120.7 (3)
C1—N1—C5	110.43 (12)	C11—C10—H10	119.6
C4—N1—H1	111.1 (14)	C10—C11—H11	120.0
C4—N1—C5	112.28 (12)	C10—C11—C12	120.1 (2)
C5—N1—H1	105.5 (14)	C12—C11—H11	120.0
C2—N2—H2	110.0 (17)	C11—C12—H12	120.3
C2—N2—C14	110.42 (11)	C11—C12—C13	119.4 (3)
C3—N2—H2	106.5 (17)	C13—C12—H12	120.3
C3—N2—C2	108.12 (11)	C8—C13—C12	120.4 (2)
C3—N2—C14	111.93 (11)	C8—C13—H13	119.8
C14—N2—H2	109.8 (16)	C12—C13—H13	119.8
N1—C1—H1A	109.6	N2—C14—H14	106.6
N1—C1—H1B	109.6	C15—C14—N2	110.76 (12)
N1—C1—C2	110.42 (12)	C15—C14—H14	106.6
H1A—C1—H1B	108.1	C21—C14—N2	112.19 (12)
C2—C1—H1A	109.6	C21—C14—H14	106.6
C2—C1—H1B	109.6	C21—C14—C15	113.49 (12)
N2—C2—C1	111.21 (12)	C16—C15—C14	122.98 (14)
N2—C2—H2A	109.4	C20—C15—C14	118.21 (14)
N2—C2—H2B	109.4	C20—C15—C16	118.80 (15)
C1—C2—H2A	109.4	C15—C16—H16	119.5
C1—C2—H2B	109.4	C17—C16—C15	120.94 (16)
H2A—C2—H2B	108.0	C17—C16—H16	119.5
N2—C3—H3A	109.5	C16—C17—H17	121.0
N2—C3—H3B	109.5	C18—C17—C16	118.02 (17)
N2—C3—C4	110.88 (13)	C18—C17—H17	121.0
H3A—C3—H3B	108.1	F1—C18—C17	118.46 (19)
C4—C3—H3A	109.5	F1—C18—C19	118.21 (18)
C4—C3—H3B	109.5	C17—C18—C19	123.32 (17)
N1—C4—C3	111.06 (12)	C18—C19—H19	121.1
N1—C4—H4A	109.4	C18—C19—C20	117.90 (17)
N1—C4—H4B	109.4	C20—C19—H19	121.1
C3—C4—H4A	109.4	C15—C20—C19	121.03 (17)
C3—C4—H4B	109.4	C15—C20—H20	119.5
H4A—C4—H4B	108.0	C19—C20—H20	119.5
N1—C5—H5A	109.4	C22—C21—C14	117.74 (14)
N1—C5—H5B	109.4	C22—C21—C26	118.70 (15)
H5A—C5—H5B	108.0	C26—C21—C14	123.46 (14)
C6—C5—N1	111.30 (13)	C21—C22—H22	119.6
C6—C5—H5A	109.4	C21—C22—C23	120.81 (17)
C6—C5—H5B	109.4	C23—C22—H22	119.6
C5—C6—H6	117.7	C22—C23—H23	120.8
C7—C6—C5	124.69 (16)	C24—C23—C22	118.38 (17)
C7—C6—H6	117.7	C24—C23—H23	120.8
C6—C7—H7	115.9	F2—C24—C23	119.28 (17)
C6—C7—C8	128.16 (17)	F2—C24—C25	117.85 (17)
C8—C7—H7	115.9	C25—C24—C23	122.87 (17)
C9—C8—C7	118.3 (2)	C24—C25—H25	120.9
C13—C8—C7	123.18 (17)	C24—C25—C26	118.21 (17)
C13—C8—C9	118.4 (2)	C26—C25—H25	120.9
C8—C9—H9	119.6	C21—C26—H26	119.5
C10—C9—C8	120.9 (3)	C25—C26—C21	121.02 (17)
C10—C9—H9	119.6	C25—C26—H26	119.5
			
F1—C18—C19—C20	178.84 (18)	C9—C10—C11—C12	0.0 (4)
F2—C24—C25—C26	−179.58 (17)	C10—C11—C12—C13	0.8 (4)
N1—C1—C2—N2	59.47 (16)	C11—C12—C13—C8	−1.1 (4)
N1—C5—C6—C7	163.61 (17)	C13—C8—C9—C10	0.2 (3)
N2—C3—C4—N1	−58.67 (16)	C14—N2—C2—C1	178.60 (12)
N2—C14—C15—C16	−61.46 (18)	C14—N2—C3—C4	179.76 (11)
N2—C14—C15—C20	119.97 (15)	C14—C15—C16—C17	−179.06 (15)
N2—C14—C21—C22	−123.12 (15)	C14—C15—C20—C19	178.65 (16)
N2—C14—C21—C26	60.5 (2)	C14—C21—C22—C23	−176.00 (17)
C1—N1—C4—C3	57.27 (16)	C14—C21—C26—C25	176.01 (17)
C1—N1—C5—C6	−168.49 (13)	C15—C14—C21—C22	110.39 (16)
C2—N2—C3—C4	57.92 (15)	C15—C14—C21—C26	−65.9 (2)
C2—N2—C14—C15	−57.12 (15)	C15—C16—C17—C18	0.5 (3)
C2—N2—C14—C21	174.93 (12)	C16—C15—C20—C19	0.0 (3)
C3—N2—C2—C1	−58.64 (15)	C16—C17—C18—F1	−179.29 (16)
C3—N2—C14—C15	−177.63 (12)	C16—C17—C18—C19	0.0 (3)
C3—N2—C14—C21	54.42 (15)	C17—C18—C19—C20	−0.5 (3)
C4—N1—C1—C2	−57.40 (16)	C18—C19—C20—C15	0.4 (3)
C4—N1—C5—C6	69.16 (16)	C20—C15—C16—C17	−0.5 (2)
C5—N1—C1—C2	178.55 (12)	C21—C14—C15—C16	65.78 (19)
C5—N1—C4—C3	−179.78 (12)	C21—C14—C15—C20	−112.80 (16)
C5—C6—C7—C8	1.9 (3)	C21—C22—C23—C24	−0.4 (3)
C6—C7—C8—C9	−141.2 (2)	C22—C21—C26—C25	−0.3 (3)
C6—C7—C8—C13	41.5 (3)	C22—C23—C24—F2	179.79 (18)
C7—C8—C9—C10	−177.3 (2)	C22—C23—C24—C25	0.1 (3)
C7—C8—C13—C12	178.0 (2)	C23—C24—C25—C26	0.1 (3)
C8—C9—C10—C11	−0.5 (4)	C24—C25—C26—C21	0.0 (3)
C9—C8—C13—C12	0.7 (3)	C26—C21—C22—C23	0.5 (3)

### Hydrogen-bond geometry (Å, º)

**Table:**

*D*—H···*A*	*D*—H	H···*A*	*D*···*A*	*D*—H···*A*
N1—H1···Cl1^i^	0.84 (2)	2.14 (2)	2.9782 (13)	176 (2)
N2—H2···Cl2^ii^	0.86 (3)	2.17 (3)	3.0248 (13)	174 (2)
C1—H1*A*···O1*W*^i^	0.99	2.68	3.512 (2)	142
C5—H5*A*···O1*W*^i^	0.99	2.67	3.470 (2)	138
C5—H5*B*···Cl1^ii^	0.99	2.51	3.4934 (16)	176
C14—H14···Cl2^i^	1.00	2.49	3.4735 (15)	168
C19—H19···F2^iii^	0.95	2.46	3.244 (2)	140
O1*W*—H1*W*···Cl1	0.80 (3)	2.48 (3)	3.2603 (12)	163 (3)

Symmetry codes: (i) −*x*+1, −*y*+2, −*z*+1; (ii) −*x*+1, −*y*+1, −*z*+1; (iii) *x*, −*y*+2, *z*+1/2.
